# Dynamic changes of secondary metabolites and tyrosinase activity of *Malus pumila* flowers

**DOI:** 10.1186/s13065-019-0602-y

**Published:** 2019-07-09

**Authors:** Lili Cui, Xingzi Hou, Wenjing Li, Yuchun Leng, Yang Zhang, Xinjuan Li, Yangyang Hou, Zhenhua Liu, Wenyi Kang

**Affiliations:** 1 National R & D Center for Edible Fungus Processing Technology, Henan University, Kaifeng, 475004 China; 2 Joint International Research Laboratory of Food & Medicine Resource Function, Kaifeng, 475004 Henan China

**Keywords:** *Malus pumila* flowers, Content change, Tyrosinase

## Abstract

The dynamic changes of secondary metabolites and tyrosinase activity of *Malus pumila* flowers were investigated during blooming for full use of *M. pumila* flowers. Phlorizin, astragalin and afzelin were selected as the indicators to determine the optimum extraction conditions of *M. pumila* flowers by HPLC analysis and three-factor and three-level orthogonal design experiments. When the smashing mesh number was 40 mesh, the ultrasonic time was 30 min, the solid–liquid ratio was 1:120, and the centrifugal speed was 10,000 r/min, the total content of the three compounds was the highest. The total content of phlorizin, astragalin and afzelin increased firstly and then decreased, and the highest was on the third day (176.74 mg/g). At the same time, the tyrosinase activity of *M. pumila* flowers showed that it had an activation effect on tyrosinase during early blooming period, however it exhibited inhibitory effect during late blooming period.

**Electronic supplementary material:**

The online version of this article (10.1186/s13065-019-0602-y) contains supplementary material, which is available to authorized users.

## Introduction

*Malus pumila* Mill has a long history of cultivation and is widely cultivated in temperate regions of the world [1]. The chemical constituents of *M. pumila* include flavonoids, terpenoids and organic acids [2–4], Which have been reported to excrete several pharmacological activities such as being antioxidant, anti-aging [5], anti-cancer [6], anti-bacterial [7], hypoglycemic [8], liver protective. *M. pumila* flowers can be used as tea [9]. Its main chemical components are dihydrochalcone such as phlorizin, phloretin, and other flavonoids such as quercetin, kaempferol and rutin [10]. In our previous study, nine compounds were isolated from *M. pumila* flowers, which exhibited activation or inhibition of tyrosinase [11]. In addition, we summarized the chemical compositions of different parts from *M. pumila* [12–14] as shown in Table 1.

**Table 1:** Compounds from different parts of *M. pumila*

Different parts	Chemical compounds	Literatures
Fruit	Proanthocyanidin B1, catechin, chlorogenic acid, proanthocyanidin B2, epicatechin, phloridzin, *β*-sitosterol, caffeic acid, phloretin, hyperoside, quercetin, quercitrin, isoquercitrin	[12, 13]
Peel	Gallic acid, protocatechuic acid, cianidanol, chlorogenic acid, caffeic acid, epicatechin, syringic acid, taxifolin, ferulic acid, quercitrin, phloridzin, quercetin	[13]
Branches	Phloridzin, phloretin	[15]
Leave	Phloridzin, phloretin, quercetin-3-*O*-glucoside, phloretin, phloridzin, quercitrin, quercetin-3-*O*-xylopyranoside	[14, 15]
Flower	Kaempferol-3-*O*-*β*-d-glucopyranoside, kaempferol-7-*O*-*β*-d-glucopyranoside, kaempferol-3-*O*-α-l-arabinofuranoside, phloridzin, kaempferol, phloretin, *β*-sitosterol, lupeol, pyracanthoside	[11]

Zhao et al. [15] reported that the content change of phlorizin was different in branches and leaves of *M. pumila*, phloretin was exclusively detected in the leaves. Tang [16] found that the main polyphenols in mature apples included catechins, proanthocyanidins and chlorogenic acids, while immature apples had mainly dihydrochalcone and flavonols. Renard et al. [17] reported that the content of proanthocyanidins increased continuously during the whole growth period of apples. Polyphenols began to synthesize 40 days after flowering. The content of flavonoids decreased significantly in the range of 35–100 days after flowering.

At present, the dynamic change of *M. pumila* is mainly focused on fruits, leaves and branches. However, there are fewer investigations on the dynamic change of *M. pumila* flowers, except for the dynamic changes of amino acids and protein [18]. In order to make full use of *M. pumila* resources, the dynamic changes of secondary metabolites and tyrosinase activity were investigated during *M. pumila* flowers blooming.

## Methods

### Chemicals and materials

Methanol was purchased from Tianjin DaMao Chemical Reagent Factory (Tianjin, China). Acetic acid was obtained from Tianjin FuChen Chemical Reagent Factory (Tianjin, China). Astragalin with purity greater than 98% was purchased from Chengdu Pufei De Biotech Co., Ltd. (Chengdu, China). Phlorizin and afzelin with purity greater than 98% were isolated in our previous research.

The LC-20AT high performance liquid chromatography system (Shimadzu, Kyoto, Japan) equipped with a degasser, a quaternary gradient low pressure pump, the CTO-20A column oven, a SPD-M20AUV-detector, an SIL-20A auto sampler was used. Chromatographic separation was performed on an Agilent ZORBAX SB-C18 column (4.6 mm × 5 mm, 5 μm) and extraction was carried out with KQ-500DB ultrasonic cleaner (Jiangsu Kunshan Ultrasonic Instrument Co., Ltd. Jiangsu, China). TGL-16 type high speed centrifuge was obtained from Jiangsu Jintan Zhongda instrument factory (Jiangsu, China). AB135-S 1/10 million electronic balance was purchased from Mettler Toledo Instruments Co., Ltd (Shanghai, China).

### Plant materials

The *M. pumila* flowers were collected during the period from March 26th 2018 to April 3rd 2018 in the campus of Henan University (Kaifeng, Henan, China) and identified by Professor Changqin Li of National R & D Center for Edible Fungus Processing Technology. They were collected once a day on nine batches. Specimens (2018-0326-0403) were deposited in the National R & D Center for Edible Fungus Processing Technology.

## Experimental methods

### Preparation of the standard solution

The standard concentrations of phlorizin, astragalin and afzelin were prepared at concentration of 0.2040, 0.1960 and 0.1990 mg/mL with methanol.

### Preparation of test sample solution

*Malus pumila* flowers powder (20.00 mg) was dissolved with appropriate solvent. The sample was extracted by ultrasound for 30 min and centrifugation for 3 min at 8000 r/min. The supernatant was filtered by 0.22 μm microporous membrane and the subsequent filtrate was taken as the test solution.

The solvent type, extraction concentration, particle size, sample-solvent ratio, ultrasonic time and centrifugal speed were investigated in turn. Every experiment was carried out in parallel three times.

### Chromatographic conditions

**Fig. 1:**
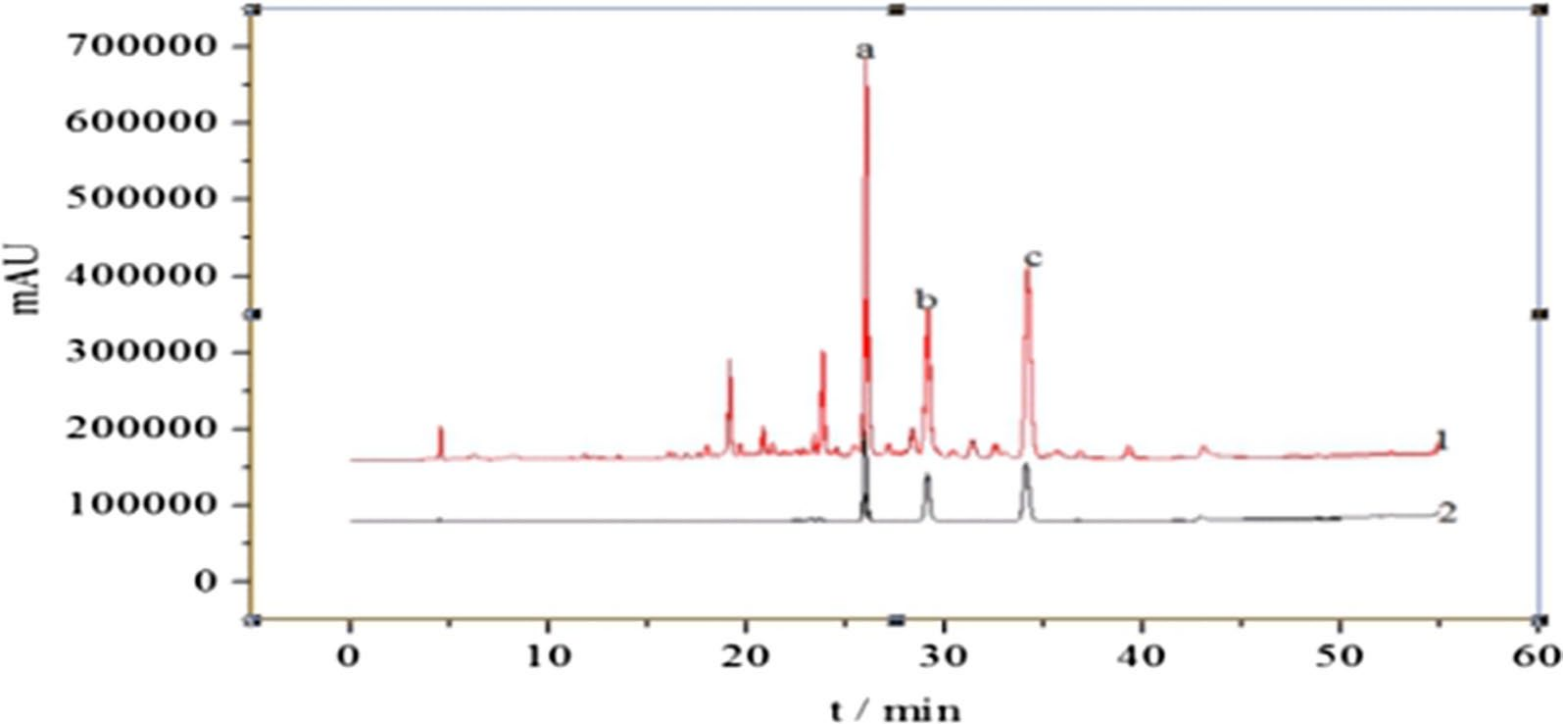
HPLC chromatograms of the test sample solution (1) and the standard solution (2): a. Phlorizin, b. Astragalin, c. Afzelin

See Table 2 and Fig. 1.

**Table 2:** Chromatographic conditions for *M. pumila* flowers samples analysis

Chromatographic conditions	Parameter
Column	Inertsil ODS-SP column (4.6 mm × 250 mm, 5 μm)
Mobile phase	Methanol (A)-0.1% phosphoric acid aqueous solution (B) 0–10 min, 5–50%A, 95–50%B 10–30 min, 50–50%A, 50–50%B 30–40 min, 50–100%A, 50–0%B 40–55 min, 100–100%A, 0–0%
Flow rate	0.8 mL/min
Column temperature	30 °C
Detection wavelength	270 nm
Sample volume	10 μL

### Tyrosinase activity assay

The absorbance was measured at 492 nm with a microplate reader, and the results were evaluated through the following formula [19]:

$${\text{Tyrosinase inhibition rate }}\% = \left[ {\frac{{{\text{A}}_{{({\text{sample}} + {\text{substrate}} + {\text{enzyme}})}} - {\text{A}}_{{({\text{sample}} + {\text{substrate}})}} }}{{{\text{A}}_{{({\text{methanol}} + {\text{substrate}} + {\text{enzyme}})}} - {\text{A}}_{{({\text{methanol}} + {\text{substrate}})}} }}} \right] - 1*100\%$$

## Results and discussion

### Linear relationship

The peak area (*X*) is the vertical axis, and the sample quality (*Y*, μg) the abscissa, respectively. In Table 3, phlorizin, astragalin and afzelin exhibited good linearity in the ranges of 0.4080–14.28 (μg/mL), 0.3920–13.72 (μg/mL) and 0.3980–13.93 (μg/mL), respectively.

**Table 3:** Linear regression equation of phlorizin, astragalin and afzelin (Additional file 1: Table S1–S3)

Compound	Regression equation	*r*	Linear range (μg)
Phlorizin	*Y *= 643736*X *+ 158998	0.9987	0.4080–14.28
Astragalin	*Y *= 518051*X *+ 145776	0.9979	0.3920–13.72
Afzelin	*Y *= 835408*X* − 359973	0.9942	0.3980–13.93

### Optimization extraction process of flavonoids in *M. pumila* flowers

#### Single factor test

##### Types of extract solvent

**Fig. 2:**
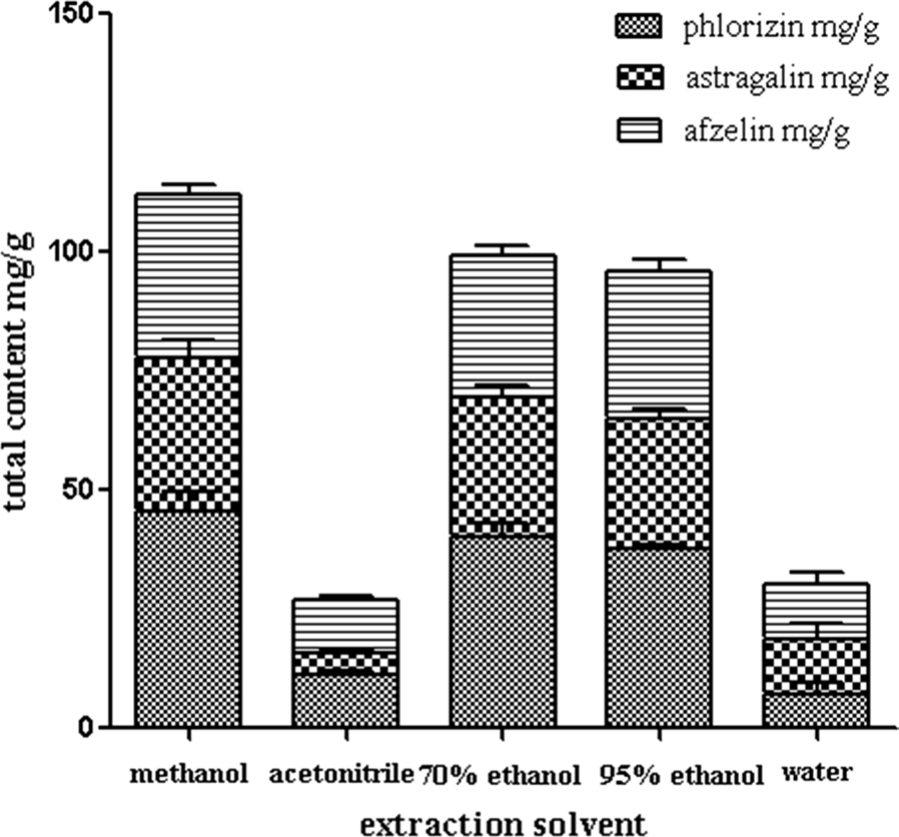
Influence of different extract solvents (*n *= 3) (Additional file 1: Table S4)

Five solvents, 70% ethanol, 95% ethanol, methanol, acetonitrile and water were selected. Samples were prepared according to the conditions in “Preparation of test sample solution” section above and injected into the HPLC analysis by above chromatographic conditions (Table 2). The results showed that acetonitrile could only extract two kinds of flavonoids from *M. pumila* flowers (Fig. 2), whereas methanol was the best solvent.

##### Selection of mesh number

**Fig. 3:**
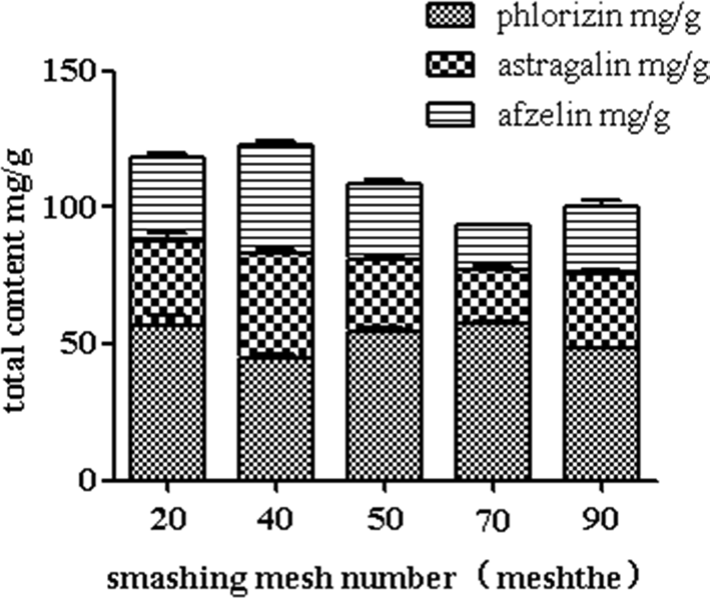
Effect of mesh numbers on extract yield (*n *= 3) (Additional file 1: Table S5)

In Fig. 3, the extract rate of target analytes was the highest when the number of smashing mesh was 40 meshes, but the extract rate was lower when the number of smashing mesh was larger. So, 40 meshes were selected.

##### Effect of ultrasonic time

**Fig. 4:**
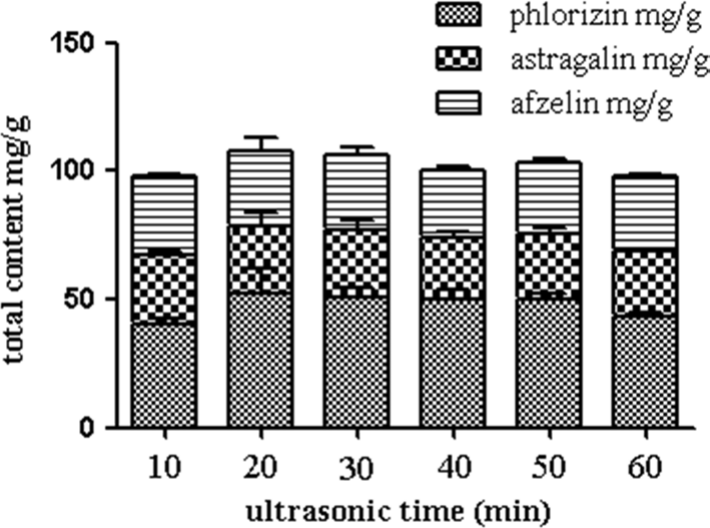
Effect of ultrasonic times on extract yield (*n *= 3) (Additional file 1: Table S6)

The ultrasonic time of 10, 20, 30, 40, 50 and 60 min were chosen respectively. According to the above experimental conditions, results were showed in Fig. 4. When the ultrasonic time was 20 min, the extract rate of the target analytes reached the maximum. With the increase of time, the target analytes extraction rate showed a downward trend, which might be the decomposition of effective components due to ultrasonic overheating [20, 21]. Hence, 20 min was selected.

##### Selection of centrifugal speed

**Fig. 5:**
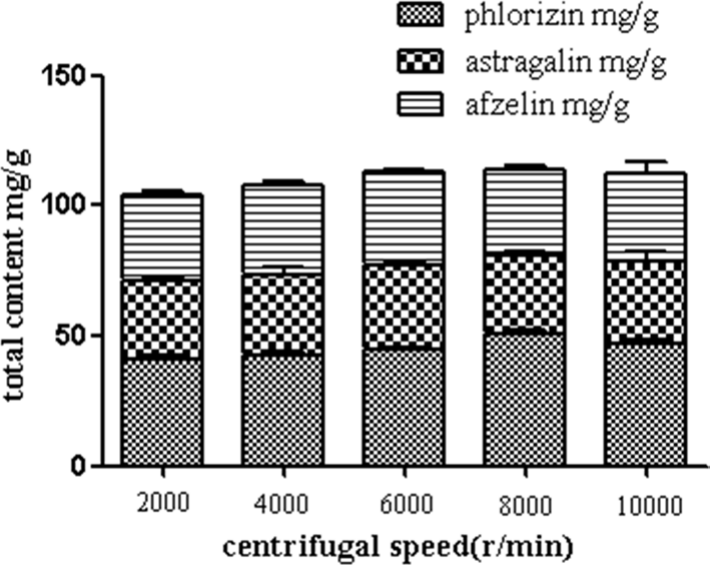
Effect of centrifugal speeds on extract yield (*n *= 3) (Additional file 1: Table S7)

Under the optimal conditions, five different centrifugal speeds (2000, 4000, 6000, 8000 and 10,000 r/min) were chosen to evaluate the effect of centrifugal speed on the extract yield. In Fig. 5, the extract rate reached the maximum at 8000 r/min. Thus, in the experiments, 8000 r/min was chosen as the center point of the orthogonal factors.

##### Sample-solvent ratio

**Fig. 6:**
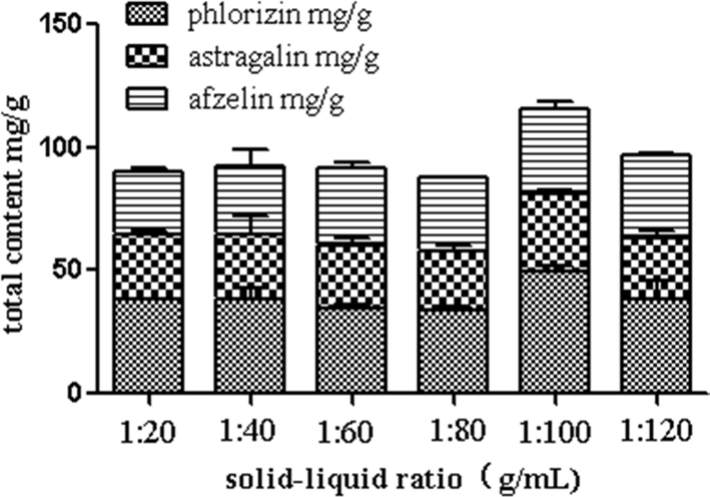
Effect of solid–liquid ratios on extract yield (*n *= 3) (Additional file 1: Table S8)

The solid–liquid ratios were 1:20, 1:40, 1:60, 1:80, 1:100 and 1:120, which were used to evaluate the extract rate of the target analytes. In Fig. 6, when the solid–liquid ratio was 1:100, the maximum extract rate was reached. Continuing to increase the ratio, the extract rate was decreased. It indicated that the increase of solvent amount can increase the contact area between the active ingredients and the solvent, and the dissolution probability of the active ingredients will also increase accordingly in a certain range and the yield will be improved continuously. However, when the ratio of solid–liquid reaches a certain value, the degree of assistant effect of ultrasonic cavitation on plant cell rupture decreases, and the dissolution of effective components is close to saturation, the extract rate decreases [22, 23].

#### Orthogonal test (Additional file 1: Table S9)

A 3-factor 3-level orthogonal experiment was designed and statistically analyzed by SPSS 19.0 [24]. The investigated levels of each factor were selected depending on the above experiment results of the single-factor. Independent variables with three variation levels were shown in Table 4.

**Table 4:** Orthogonal test factors and level tables

Level	Factor		
	A Solid–liquid ratio (times)	B Ultrasound time (min)	C Centrifugal speed (r/min)
1	1:120	10	6000
2	1:100	20	8000
3	1:80	30	10,000

Through the orthogonal test of ultrasonic time (min), solid–liquid ratio (times) and centrifugal speed (r/min), the variance analysis was carried out by SPSS 19.0 software. The results were presented in Tables 5 and 6. The ultrasonic time had the greatest influence, followed by the solid–liquid ratio. The primary and secondary sequence of experimental factors were: (B) ultrasonic time > (A) solid–liquid ratio > (C) centrifugal speed. A1B3C3 was the optimal extraction process, namely: the solid–liquid ratio was 1:120, the ultrasonic time was 30 min, and the centrifugal speed was 10,000 r/min.

**Table 5:** Results of range analysis

Serial number	A	B	C	Total content mg/g
1	3	3	1	97.07
2	1	2	3	98.47
3	3	1	3	106.15
4	1	3	2	113.74
5	2	3	3	97.21
6	3	2	2	89.59
7	2	2	1	95.53
8	2	1	2	85.69
9	1	1	1	89.96
$\overline{\text{K}} 1$	100.72	93.93	94.19	
$\overline{\text{K}} 2$	92.81	94.53	96.34	
$\overline{\text{K}} 3$	97.60	102.68	100.61	
Rj	7.91	8.74	6.42

**Table 6:** Variance analysis of factors

	Sum of squares of III	df	Mean square	F	Sig.
Model	85,064.737^a^	7	12,152.105	81.537	0.012
A	95.303	2	47.651	0.32	0.758
B	143.1	2	71.55	0.48	0.676
C	64.1	2	32.05	0.215	0.823
Error	298.075	2	149.037		
Total	85,362.812	9			

^a^R^2^ = 0.9970 (adjust R^2^ = 0.9840)

### Determination of content in different periods

**Fig. 7:**
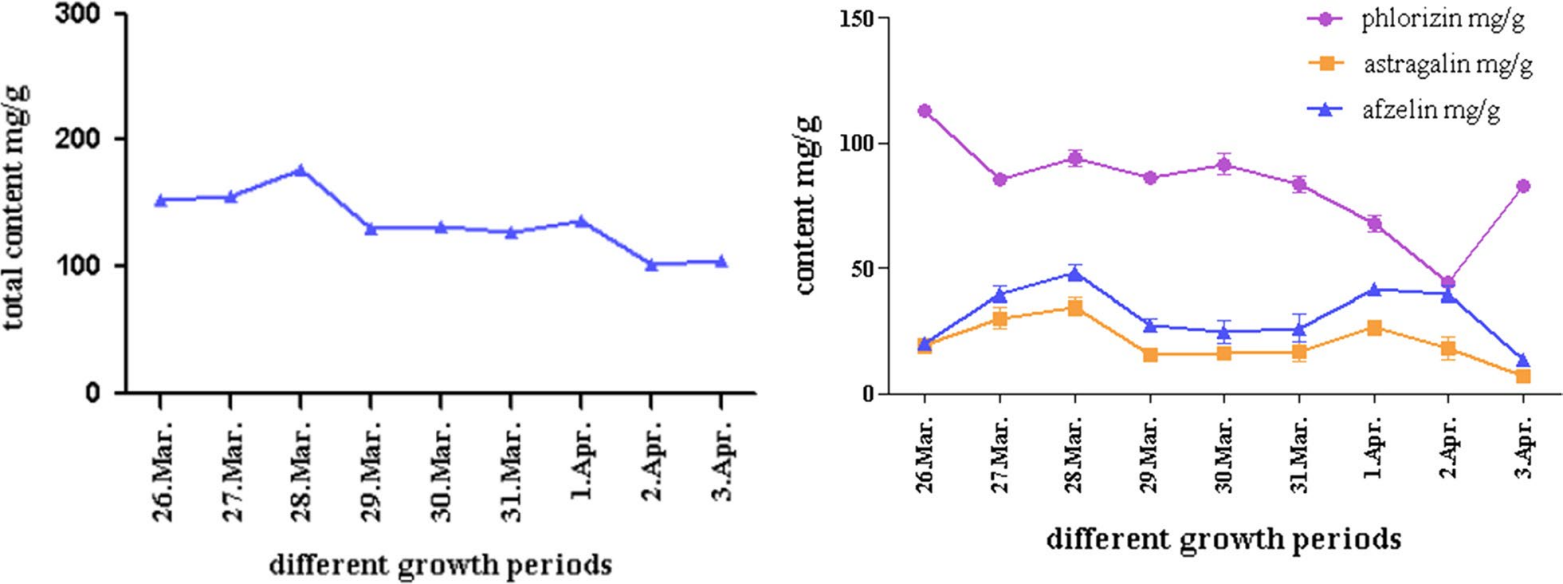
Changes of three secondary metabolites in *M. pumila* flowers (Additional file 1: Table S10)

In Fig. 7, the total content of phlorizin, astragalin and afzelin reached the highest level on the third day (176.74 mg/g) during blooming of *M. pumila* flowers. The contents of astragalin and afzelin decreased after 1st April, while the contents of phlorizin increased after 2nd April. It indicated that the phlorizin was transferred and enriched in fruits, resulting in the fruit containing a large amount of phlorizin [25]. Moreover, light intensity, soil acidity and alkalinity and external temperature also affect the formation of secondary metabolites [26].

### Method validation

#### Repeatability (Additional file 1: Table S11)

Six *M. pumila* flowers samples were prepared by “Preparation of test sample solution” method, and the contents of phlorizin, astragalin and afzelin were determined under “Chromatographic conditions” section. The results showed that RSDs of phlorizin, astragalin and afzelin were 0.92%, 1.88% and 2.17% respectively, which indicated that the method had good repeatability.

#### Precision (Additional file 1: Table S12)

The mixed standard solution of phlorizin, astragalin and afzelin was prepared under “Preparation of the standard solution” conditions. According to “Chromatographic conditions” section, mixed standard solution was injected continuously for 6 times. The results showed that RSDs of phlorizin, astragalin and afzelin were 0.8%, 1.02% and 0.75% respectively, indicating that the instrument had good precision.

#### Stability (Additional file 1: Table S13)

Six *M. pumila* flowers samples were prepared by “Preparation of test sample solution” conditions. According to “Chromatographic conditions” section, samples were injected at 0, 4, 8, 12, 16, 20 and 24 h, respectively. The results showed that RSDs of phlorizin, astragalin and afzelin were 0.35%, 0.95% and 0.84% respectively, which indicated that phlorizin, astragalin and afzelin in the samples were basically stable within 24 h.

#### Recovery (Additional file 1: Table S14)

Six samples of *M. pumila* flowers were prepared according to 3.2 conditions. The contents of phlorizin, astragalin and afzelin were determined. Then the standard solution equivalent to 80% of the three target components in the sample was added. The average recoveries of phlorizin, astragalin and afzelin were 98.20%, 98.96% and 101.03% respectively, and their RSDs values were 0.20%, 0.24% and 0.13%, respectively.

### Tyrosinase activity assay

**Fig. 8:**
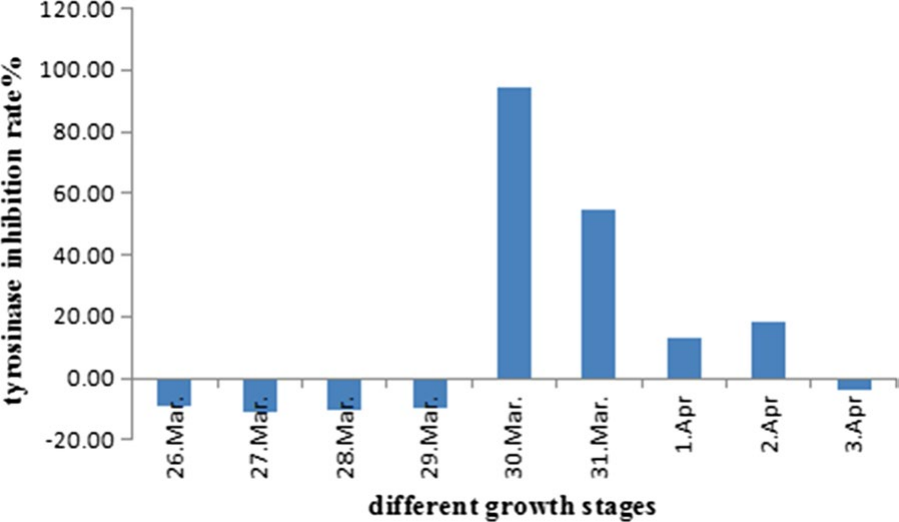
Changes of tyrosinase activity in *M. pumila* flowers during blooming

The response of tyrosinase activity was determined by tyrosinase and dopa rate oxidation trace method in vitro. In Table 7 and Fig. 8, *M. pumila* flowers could stimulate the activity of tyrosinase in the early stage of blooming. Meanwhile, inhibition in the activity of tyrosinase occured in the late stage of blooming, which was gradually decreased. The reason may be that in the late stage of blooming, the content of secondary metabolites which inhibit the activity of tyrosinase increased, so the total extracts of *M. pumila* flowers showed inhibition activity of tyrosinase.

**Table 7:** Changes of tyrosinase activity in *M. pumila* flowers during blooming ($\overline{\text{X}} \pm {\text{s}}$)

Collecting time	Inhibition rate %
26th March	− 9.21 ± 2.68
27th March	− 10.91 ± 7.73
28th March	− 10.63 ± 7.75
29th March	− 9.47 ± 3.40
30th March	94.33 ± 0.72
31th March	54.47 ± 4.57
1st April	13.08 ± 1.89
2nd April	18.36 ± 2.52
3rd April	− 4.01 ± 7.13

Tyrosinase is a key enzyme in melanin synthesis, and its activity is positively correlated with the amount of melanin [27, 28]. It is well known that melanin protects the skin from UV damage but its excessive production causes freckles, melasma, skin cancer, and age spots [29, 30]. Xie et al. [31] studied the inhibition kinetics of flavonoids on mushroom tyrosinase and suggested that flavonoids could induce reversible inhibition of enzyme activity through copper ions in the active center of chelating enzyme. Beside the effect of flavonoids, there might be other tyrosinase inhibitors, which could bind to free enzymes as well. It can bind with enzyme–substrate complex, resulting in reversible inhibition of enzyme activity. Several studies have shown that many flower extracts had good tyrosinase activity, such as orchid extract [32], lavender essential oil [33], areca nut boiling water extracts [34], and can be used as effective tyrosinase inhibitors. At present, there were few reports about the effect of *M. pumila* flowers on tyrosinase activity. The obtained results indicated that the content of three flavonoids reached the highest on 28th March during blooming of *M. pumila* flowers. The methanol extracts of the flowers picked on 30th March had a higher inhibitory effect on tyrosinase, which may advocated that tyrosinase activity not only related to flavonoids, but also related to the types of secondary metabolites extracted as well as the mechanism of action.

## Conclusion

Under optimum extraction conditions (smashing mesh number: 40 meshes, ultrasonic time: 30 min, solid–liquid ratio: 1:120, centrifugal speed: 10,000 r/min), the total contents of phlorizin, astragalin and afzelin from *M. pumila* flowers reached the maximum (176.74 mg/g) on the third day of blooming, which could be the best time for harvest. Meanwhile, the tyrosinase activity of *M. pumila* flowers showed that it had an activation effect on tyrosinase during early blooming period, however it expressed inhibitory effect during late blooming period.

## Additional file

**Additional file 1.** **Table S1–S3.** The raw data for Table 3 Linear regression equation of phlorizin, astragalin and afzelin in the manuscript. **Table S4.** The raw data for Fig. 2 Influence of different extract solvents in the manuscript. **Table S5.** The raw data for Fig. 3 Effect of mesh numbers on extract yield in the manuscript. **Table S6.** The raw data for Fig. 4 Effect of ultrasonic times on extract yield in the manuscript. **Table S7.** The raw data for Fig. 5 Effect of centrifugal speeds on extract yield in the manuscript. **Table S8.** The raw data for Fig. 6 Effect of solid-liquid ratios on extract yield in the manuscript. **Table S9.** The raw data for orthogonal test. **Table S10.** The raw data for Fig. 7 Changes of three secondary metabolites in *M. pumila* flowers in the manuscript. **Table S11.** The raw data for 4.5.1 Repeatability experiment in the manuscript. **Table S12.** The raw data for 4.5.2 Precision experiment in the manuscript. **Table S13.** The raw data for 4.5.3 Stability experiment in the manuscript. **Table S14.** The raw data for 4.5.4 Recovery experiment in the manuscript.

## Data Availability

The datasets and samples of the compounds are available from the authors.

## Abbreviations

*M. pumila* flowers: *Malus pumila* flowers
HPLC: high-performance liquid chromatography
Fig: figure
RSD: relative standard deviation
Mar: March
Apr: April
