# Supplementary material for: Dynamic changes of secondary metabolites and tyrosinase activity of Malus pumila flowers
Source: BMC Chem. 2019 Jul 9;13(1):81. doi: 10.1186/s13065-019-0602-y (PMC6661837; doi:10.1186/s13065-019-0602-y)
Supplement: Supplementary file 1 — Additional file 1. Table S1–S3. The raw data for Table 3 Linear regression equation of phlorizin, astragalin and afzelin in the manuscript. Table S4. The raw data for Fig. 2 Influence of different extract solvents in the manuscript. Table S5. The raw data for Fig. 3 Effect of mesh numbers on extract yield in the manuscript. Table S6. The raw data for Fig. 4 Effect of ultrasonic times on extract yield in the manuscript. Table S7. The raw data for Fig. 5 Effect of centrifugal speeds on extract yield in the manuscript. Table S8. The raw data for Fig. 6 Effect of solid-liquid ratios on extract yield in the manuscript. Table S9. The raw data for orthogonal test. Table S10. The raw data for Fig. 7 Changes of three secondary metabolites in M. pumila flowers in the manuscript. Table S11. The raw data for 4.5.1 Repeatability experiment in the manuscript. Table S12. The raw data for 4.5.2 Precision experiment in the manuscript. Table S13. The raw data for 4.5.3 Stability experiment in the manuscript. Table S14. The raw data for 4.5.4 Recovery experiment in the manuscript. [file 13065_2019_602_MOESM1_ESM.docx]

**Additional file**

**Dynamic changes of secondary metabolites and tyrosinase activity of *Malus pumila* flowers**

Lili Cui^1,2‡^, Xingzi Hou^1,2‡^, Wenjing Li^1,2^, Yuchun Leng^1^, Yang Zhang^1^, Xinjuan Li^1^, Yangyang Hou^1^, Zhenhua Liu^1,2*^, Kang Wenyi^1,2*^

1. National R & D Center for Edible Fungus Processing Technology, Henan University, Kaifeng 475004, China

2. Joint International Research Laboratory of Food & Medicine Resource Function, Henan Province, Kaifeng 475004, China

^‡^These authors contributed equally to this work.

*Correspondence to: Zhenhua Liu, email: liuzhenhua623@163.com; Wenyi Kang, email: kangweny@hotmail.com

**Contents of Additional file**

| No. | **Contents** | **Page** |
| --- | --- | --- |
| 1. | Table S1 Linear regression equation of phlorizin | 1 |
| 2. | Table S2. Linear regression equation of astragalin | 1 |
| 3. | Table S3. Linear regression equation of afzelin | 1 |
| 4. | Table S4 Influence of different extract solvents | 2 |
| 5. | Table S5 Effect of mesh numbers on extract yield | 2 |
| 6. | Table S6 Effect of ultrasonic times on extract yield | 2 |
| 7. | Table S7 Effect of centrifugal speeds on extract yield | 3 |
| 8. | Table S8 Effect of solid-liquid ratios on extract yield | 3 |
| 9. | Table S9 Results of orthogonal test | 3 |
| 10. | Table S10 Changes of three secondary metabolites in *M. pumila* flowers | 4 |
| 11. | Table S11 Repeatability | 4 |
| 12. | Table S12 Precision | 4 |
| 13. | Table S13 Stability | 5 |
| 14. | Table S14 Recovery | 5 |

Table S1 Linear regression equation of phlorizin

| vertical axis | abscissa |
| --- | --- |
| 0.408 | 278178 |
| 1.02 | 696905 |
| 2.04 | 1456942 |
| 4.08 | 2874018 |
| 6.12 | 4283536 |
| 8.16 | 5732235 |
| 10.2 | 6590483 |
| 12.24 | 8002930 |
| 14.28 | 9205207 |

Table S2. Linear regression equation of astragalin

| vertical axis | abscissa |
| --- | --- |
| 0.392 | 212829 |
| 0.98 | 531150 |
| 1.96 | 1144884 |
| 3.92 | 2266940 |
| 5.88 | 3387788 |
| 7.84 | 4511842 |
| 9.8 | 5092244 |
| 11.76 | 6167584 |
| 13.72 | 7138206 |

Table S3. Linear regression equation of afzelin

| vertical axis | abscissa |
| --- | --- |
| 0.398 | 315088 |
| 0.995 | 794863 |
| 1.99 | 1379174 |
| 3.98 | 2732659 |
| 5.97 | 4082111 |
| 7.96 | 5437146 |
| 9.95 | 8288527 |
| 11.94 | 9950213 |
| 13.93 | 11493095 |

Table S4 Influence of different extract solvents

| solvent | phlorizinmg/g | astragalinmg/g | afzelinmg/g | total content mg/g |
| --- | --- | --- | --- | --- |
| methanol | 45.61 | 32.21 | 34.33 | 112.15 |
| acetonitrile | 11.10 | 4.52 | 11.26 | 26.88 |
| 70% ethanol | 40.30 | 29.11 | 29.78 | 99.19 |
| 95% ethanol | 37.53 | 27.44 | 31.03 | 95.99 |
| water | 7.26 | 11.44 | 11.69 | 30.40 |

Table S5 Effect of mesh numbers on extract yield

| mesh numbers | phlorizinmg/g | astragalinmg/g | afzelinmg/g | total content mg/g |
| --- | --- | --- | --- | --- |
| 20 | 56.73 | 30.65 | 30.65 | 118.02 |
| 40 | 44.89 | 37.87 | 39.95 | 122.71 |
| 50 | 54.60 | 26.35 | 27.60 | 108.55 |
| 70 | 57.40 | 19.34 | 16.58 | 93.32 |
| 90 | 48.22 | 27.24 | 24.74 | 100.21 |

Table S6 Effect of ultrasonic times on extract yield

| ultrasonic time(min) | phlorizinmg/g | astragalinmg/g | afzelinmg/g | total content mg/g |
| --- | --- | --- | --- | --- |
| 10 | 40.35 | 26.73 | 30.61 | 97.69 |
| 20 | 52.19 | 26.34 | 29.59 | 108.12 |
| 30 | 50.41 | 26.91 | 29.04 | 106.35 |
| 40 | 49.90 | 24.26 | 25.82 | 99.99 |
| 50 | 50.03 | 25.66 | 27.47 | 103.16 |
| 60 | 43.43 | 25.66 | 28.56 | 97.65 |

Table S7 Effect of centrifugal speeds on extract yield

| centrifugal speed(r/min) | phlorizinmg/g | astragalinmg/g | afzelinmg/g | total content mg/g |
| --- | --- | --- | --- | --- |
| 2000 | 40.70 | 30.63 | 32.90 | 104.23 |
| 4000 | 42.67 | 30.82 | 34.15 | 107.64 |
| 6000 | 44.93 | 32.37 | 35.48 | 112.77 |
| 8000 | 50.81 | 29.85 | 33.35 | 114.00 |
| 10000 | 46.75 | 31.47 | 33.78 | 111.99 |

Table S8 Effect of solid-liquid ratios on extract yield

| Sample-solvent ratio | phlorizinmg/g | astragalinmg/g | afzelinmg/g | total content mg/g |
| --- | --- | --- | --- | --- |
| 1：120 | 38.19 | 24.64 | 33.84 | 96.68 |
| 1：100 | 49.35 | 31.59 | 34.66 | 115.59 |
| 1：80 | 33.66 | 24.41 | 29.79 | 87.86 |
| 1：60 | 34.41 | 25.83 | 31.26 | 91.50 |
| 1：40 | 38.72 | 26.03 | 27.70 | 92.46 |
| 1：20 | 38.26 | 26.49 | 25.67 | 90.43 |

Table S9 Results of orthogonal test

| peak area | phlorizin | astragalin | afzelin | phlorizinmg/g | astragalinmg/g | afzelinmg/g | total content mg/g |
| --- | --- | --- | --- | --- | --- | --- | --- |
| 1 | 3740335 | 1718900 | 2601046 | 44.3139 | 24.2824 | 28.4779 | 97.0742 |
| 2 | 2707104 | 1088643 | 1630616 | 47.4278 | 21.9892 | 29.0509 | 98.4679 |
| 3 | 3714309 | 1937223 | 3243965 | 43.9928 | 27.6239 | 34.5343 | 106.1510 |
| 4 | 3063623 | 1272489 | 1895897 | 54.3604 | 26.3744 | 33.0027 | 113.7374 |
| 5 | 2671349 | 1453334 | 2401321 | 38.8239 | 25.2171 | 33.1730 | 97.2140 |
| 6 | 3443619 | 1614357 | 2357358 | 40.6861 | 22.7004 | 26.2032 | 89.5897 |
| 7 | 2571585 | 1498695 | 2339240 | 37.1782 | 26.0055 | 32.3472 | 95.5309 |
| 8 | 2467545 | 1325419 | 1996526 | 35.1988 | 22.4629 | 28.0273 | 85.6890 |
| 9 | 1855969 | 1242916 | 1886103 | 31.6915 | 25.5672 | 32.7058 | 89.9645 |

Table S10 Changes of three secondary metabolites in *M. pumila* flowers

| different growth periods | phlorizinmg/g | astragalin mg/g | afzelinmg/g | total content mg/g |
| --- | --- | --- | --- | --- |
| 26^th^ March | 112.74 | 19.09 | 20.29 | 152.13 |
| 27^th^ March | 85.19 | 29.93 | 39.71 | 154.83 |
| 28^th^ March | 93.92 | 34.71 | 48.11 | 176.74 |
| 29^th^ March | 86.33 | 15.66 | 27.41 | 129.4 |
| 30^th^ March | 91.38 | 15.98 | 24.68 | 132.04 |
| 31^th^ March | 83.36 | 16.86 | 26.25 | 126.46 |
| 1^st^ April | 67.78 | 26.27 | 41.49 | 135.53 |
| 2^nd^ April | 44.31 | 18.08 | 39.65 | 102.03 |
| 3^rd^ April | 83.08 | 7.24 | 13.53 | 103.84 |

Table S11 Repeatability

| peak area | phlorizin | astragalin | afzelin |
| --- | --- | --- | --- |
| 1 | 673479 | 199060 | 190911 |
| 2 | 669668 | 197780 | 203223 |
| 3 | 680885 | 204349 | 200226 |
| 4 | 663200 | 194201 | 196363 |
| 5 | 668656 | 202682 | 198159 |
| 6 | 675794 | 197065 | 195013 |
| sd | 6157.42 | 3747.53 | 4273.35 |
| ave | 671947.00 | 199189.50 | 197315.83 |
| rsd% | 0.91635 | 1.88139 | 2.16574 |

Table S12 Precision

| peak area | phlorizin | astragalin | afzelin |
| --- | --- | --- | --- |
|  | 5873346 | 3165953 | 3737029 |
|  | 5962061 | 3208470 | 3799508 |
|  | 5982244 | 3218833 | 3811752 |
|  | 5973499 | 3196136 | 3813546 |
|  | 5908002 | 3135392 | 3795955 |
|  | 5995350 | 3213498 | 3803518 |
| ave | 5949083 | 3189714 | 3793551 |
| sd | 47809 | 32625 | 28518 |
| rsd% | 0.80 | 1.02 | 0.75 |

Table S13 Stability

| peak area | phlorizin | astragalin | afzelin |
| --- | --- | --- | --- |
|  | 6738581 | 2653643 | 3249987 |
|  | 6737100 | 2653880 | 3242424 |
|  | 6781107 | 2698094 | 3293239 |
|  | 6780611 | 2706099 | 3302594 |
|  | 6783143 | 2694055 | 3291606 |
| sd | 24003.67694 | 25379.01999 | 27622.41227 |
| ave | 6764108.4 | 2681154.2 | 3275970 |
| rsd% | 0.354868307 | 0.946570697 | 0.843182699 |

Table S14 Recovery

| peak area | phlorizin | astragalin | afzelin | phlorizinug/mg | astragalin ug/mg | afzelinug/mg |
| --- | --- | --- | --- | --- | --- | --- |
|  | 1228073 | 271952 | 1353520 | 8.2007 | 1.3688 | 10.5570 |
|  | 790851 | 233632 | 1762238 | 4.8808 | 0.9929 | 6.9570 |
|  | 1051613 | 267528 | 1395459 | 6.8608 | 1.3254 | 5.7813 |
|  | 1021544 | 248548 | 1359174 | 6.6325 | 1.1392 | 5.5655 |
|  | 891398 | 236200 | 1392239 | 5.6442 | 1.0181 | 5.7621 |
| Amount of addition | 0.712 | 0.0896 | 0.5800 | 14.2400 | 1.7900 | 11.6000 |
|  | 0.36 | 0.0544 | 0.6150 | 7.2000 | 1.0880 | 12.3040 |
|  | 0.69 | 0.1220 | 0.5950 | 13.8420 | 2.4480 | 11.9040 |
|  | 0.577 | 0.0950 | 0.6380 | 12.7680 | 1.9040 | 11.5360 |
|  | 0.614 | 0.1050 | 0.5870 | 12.2880 | 2.0960 | 11.7440 |
| ave | 0.5906 | 0.0932 | 0.603 | 12.0676 | 1.8652 | 11.8176 |
| Recovery | 0.9820 | 0.9896 | 1.0603 |  |  |  |
